# Assessment of the Role of Free-Living and Farmed Fallow Deer (*Dama dama*) as A Potential Source of Human Infection with Multiple-Drug-Resistant Strains of *Yersinia enterocolitica* and *Yersinia pseudotuberculosis*

**DOI:** 10.3390/pathogens11111266

**Published:** 2022-10-30

**Authors:** Marta Odyniec, Agata Bancerz-Kisiel

**Affiliations:** Department of Epizootiology, Faculty of Veterinary Medicine, University of Warmia and Mazury, Oczapowskiego 2 St., 10-719 Olsztyn, Poland

**Keywords:** *Yersinia enterocolitica*, *Yersinia pseudotuberculosis*, free-living fallow deer, farmed fallow deer, multiple drug resistance

## Abstract

*Yersinia enterocolitica* and *Y. pseudotuberculosis* are Gram-negative, facultative anaerobic bacteria that cause yersiniosis—one of the most important zoonotic diseases of the digestive tract. The aim of this study was to determine the prevalence of potentially human-pathogenic *Y. enterocolitica* and *Y. pseudotuberculosis* strains in free-living and farmed fallow deer, and to evaluate their sensitivity to chemotherapeutics. A total of 372 rectal swabs were analyzed, including 262 from free-living and 110 from farmed fallow deer. Due to the psychrophilic properties of *Yersinia*, two samples were collected from each animal. Seven *Y. enterocolitica* strains were isolated from free-living fallow deer, while two strains were isolated from farmed fallow deer. *Yersinia pseudotuberculosis* strains were not identified. All isolated *Y. enterocolitica* strains were *ystB*-positive, and phylogenetic analysis based on the nucleotide sequences of this gene revealed the presence of two phylogenetic groups. *Yersinia enterocolitica* strains isolated from fallow deer belonged to biotype 1A, and serotyping analysis demonstrated that the vast majority did not agglutinate with any diagnostic sera. All strains were multiple drug resistant and were not sensitive to at least four of the tested chemotherapeutics (amoxicillin with clavulanic acid, ampicillin, cefalexin, and streptomycin). One *Y. enterocolitica* strain isolated from a free-living animal was resistant to nine out of the 13 analyzed chemotherapeutics and was intermediately sensitive to the four remaining chemotherapeutics. The highest sensitivity was noted in case of ciprofloxacin (five strains) and trimethoprim-sulfamethoxazole (three strains). Only one strain isolated from a free-living animal was sensitive to three out of the 13 examined antibiotics, whereas the remaining strains were sensitive to only one drug or were not sensitive to any of the chemotherapeutics used. The results of this study indicate that multiple drug-resistant *Y. enterocolitica* strains can be carried by free-living and farmed fallow deer. This observation gives serious cause for concern because the meat of fallow deer and other ruminants is often consumed semi-raw (steak) or raw (tartar steak).

## 1. Introduction

Within the *Yersinia* genus, three species are human pathogens: *Y. pestis*, which is the causative agent of plague, and two enteropathogens, *Y. pseudotuberculosis* and *Y. enterocolitica*, which are transmitted by ingestion of contaminated food [1]. The European Food Safety Authority (EFSA) classifies yersiniosis as one of the most important zoonotic diseases of the digestive tract. In the latest report, yersiniosis was the fourth most frequently reported zoonosis in humans in 2019 [2]. *Yersinia enterocolitica* and *Y. pseudotuberculosis* are both Gram-negative, facultative anaerobic bacteria with a size of 0.5–2 µm [3]. The optimal temperature for their growth is 22–29 °C, but these psychrophilic bacteria can also proliferate at low temperatures (0–4 °C), which enables them to survive in unsupportive environments [4,5]. 

The epidemiology of infections caused by *Y. enterocolitica* and *Y. pseudotuberculosis* is highly complex. Livestock, mainly pigs, are a reservoir of *Y. enterocolitica*. A strong correlation was reported between the bioserotypes of strains isolated from humans and pigs in the same geographic region [6,7]. Moreover, DNA analyses revealed high phylogenetic similarity between strains that are pathogenic for humans and strains isolated from pigs [8,9]. Meanwhile, small rodents and wild birds have been regarded as the main reservoir of human-pathogenic *Y. pseudotuberculosis* [10,11,12]. However, there is considerable evidence to indicate that *Y. enterocolitica* and *Y. pseudotuberculosis* both colonize numerous species of free-living animals [13,14,15,16,17,18,19,20,21,22,23,24]. The environmental impact of infected free-living animals on other animal species’ welfare, as well as that of humans, is on the rise.

These observations are particularly important in view of the growing consumption of game meat [25]. Fallow deer (*Dama dama*) carcasses are characterized by a high proportion of lean meat, a high content of minerals (in particular Fe and Cu) and a desirable fatty acid profile [25]. Therefore, fallow deer meat is a valuable component of the human diet, and deer farms are a steady source of game meat with consistently high quality [26]. The first fallow deer farm oriented towards meat production was established in New Zealand in 1969 [27]. In Poland, commercial deer farms have been in operation since 2001, and fallow deer represent the most popular species, raised in 80% of deer farms [28]. 

Animals and humans ingest bacteria of the genus *Yersinia* with food and water, and the expression of bacterial virulence markers is induced under the influence of body temperature [29]. All pathogenic strains of *Y. enterocolitica* and *Y. pseudotuberculosis* carry plasmids of 64,000–75,000 base pairs (bp) that act as antiphagocytic factors. These structures are referred to as plasmids of *Yersinia* virulence (pYV) [30]. However, the expression of pYV can be lost in *Yersinia* strains, and evaluations of pathogenicity based only on plasmid levels can produce false negative results [31]. In addition to virulence plasmids, pathogenic strains of *Y. enterocolitica* and *Y. pseudotuberculosis* also harbor genetically stable, chromosomally encoded virulence factors. These include the *inv* gene encoding the production of invasin (Inv), the *ail* gene encoding the production of the attachment invasion locus (Ail) protein, the *myf* gene encoding the production of mucoid *Yersinia* factor/fibrillae A (MyfA) protein, and the *yst* genes encoding the production of *Yersinia* stable toxins (Yst enterotoxins) [31]. 

*Yersinia enterocolitica* is a species highly diverse in terms of biotype, serotype, and potential pathogenicity. Based on their biochemical properties, *Y. enterocolitica* strains have been divided into six biotypes, i.e., 1A, 1B, 2, 3, 4, and 5 [3], which are also used for determination of their pathogenic potential. Biotype 1B is regarded as definitely pathogenic for humans and animals. Biotypes 2–5 are considered moderately pathogenic, whereas biotype 1A strains are frequently isolated from the environment and are generally regarded as non-pathogenic [32]. *Yersinia enterocolitica* strains have also been divided into more than 70 serological groups based on differences in the structure of the heat-stable somatic O antigen [33]. For many years, strains belonging to biotype 4, serotype O:3 and biotype 2, serotype O:9 were predominant in Europe, whereas strains belonging to biotype 1B, serotype O:8 were most prevalent in the United States and Japan [5]. That division is no longer apparent.

*Yersinia pseudotuberculosis* is a relatively homogeneous species that has been divided into 15 serotypes (O:1–O:15) based on the structure of the lipopolysaccharide chain of the O antigen [34]. Serotypes O:1 and O:2 have been further divided into subtypes a, b, and c, and serotypes O:4 and O:5 into subtypes a and b [35]. The remaining serotypes do not have subtypes. Serotypes O:1–O:3 have been identified mainly in Europe, serotypes O:4–O:15 mainly in Asia, and serotype O:4b is particularly prevalent in Japan [36,37,38]. All *Y. pseudotuberculosis* strains are considered pathogenic, although O:1a and O:1b are usually isolated from humans with gastroenteritis [11]. *Yersinia pseudotuberculosis* can be identified at species level based on its biochemical properties. 

The aim of this study was to determine the prevalence of potentially human-pathogenic *Y. enterocolitica* and *Y. pseudotuberculosis* strains in free-living and farmed fallow deer, to identify the bioserotype and genotype of the isolated strains, and to evaluate their sensitivity to chemotherapeutics.

## 2. Materials and Methods

### 2.1. Materials 

The material for the study consisted of rectal swabs collected from 131 free-living and 55 farmed fallow deer. Rectal swabs were collected from free-living fallow deer between fall 2016 and spring 2019, during the hunting seasons established by the Polish Hunting Association, in hunting districts throughout Poland. Two samples were taken from each animal, immediately after shooting and evisceration. Samples from farmed animals were obtained in fall or winter or in early spring, during seasonal deworming treatment. Sample collection was performed according to the Act for the Protection of Animals for Scientific or Educational Purposes of 15 January 2015 (Official Gazette 2015, No. 266), applicable in the Republic of Poland. Because samples were collected during veterinary treatment, these activities did not require additional agreement from the Bioethical Committee. Rectal samples were taken with the use of sterile swabs in test tubes without a medium (Equimed). The collected samples were transported in a refrigerator (for no longer than 5 h) to the laboratory of the Department of Epizootiology in the Faculty of Veterinary Medicine at the University of Warmia and Mazury in Olsztyn, where they were placed in the culturing media immediately after arrival.

### 2.2. Strain Isolation and Molecular Confirmation

Due to the psychrophilic properties of *Y. enterocolitica* and *Y. pseudotuberculosis*, two samples were collected from each animal and cultured on two types of media: ITC (irgasan, ticarcillin, and potassium chlorate medium; warm culture) and PSB (peptone, sorbitol, and bile salts medium; cold culture). Samples cultured on ITC were incubated at a temperature of 25 °C for 48 h, and samples cultured on PBS were incubated at a temperature of 4 °C for 3 weeks. The protocol for handling the emerged strains was identical for both types of culture. Using a sterile pipette, 0.5 mL of the culture was transferred to 4.5 mL of 0.5% KOH solution and shaken for 20 s (leaching). A culture loop was then utilized to plate 0.1 mL of the leached material on CIN (Cefsulodin-Irgasan-Novobiocin) agar to obtain single colonies. The plates were incubated at a temperature of 30 °C for 24 h, and they were evaluated under a magnifying glass with a light source. When cultured on CIN agar, *Y. enterocolitica* forms small colonies with an estimated diameter of 1 mm, smooth semi-transparent borders, and a dark red, non-opalescent center. *Yersinia pseudotuberculosis* forms red pin colonies on CIN agar. Plates that did not display characteristics of *Yersinia* colonies or where colony growth was slow or non-specific were left in the incubator for a further 24 h. 

Total genomic DNA was isolated with a genomic mini kit (A&A Biotechnology, Gdynia, Poland) for isolating DNA from bacterial cells, bacterial cultures, and solid tissues. In this method of DNA separation, DNA molecules bind to silica surfaces in the presence of high concentrations of chaotropic salts. Genomic DNA was isolated from individual colonies with phenotypic traits characteristic of *Y. enterocolitica* or *Y. pseudotuberculosis*, according to the manufacturer’s instructions. *Yersinia enterocolitica* strains were identified by amplifying fragments of *ail, ystA*, *ystB*, and *ystC* genes. A multiplex PCR method was developed to enable the identification of all four genes in a single reaction. *Yersinia pseudotuberculosis* strains were identified by amplifying fragments of *inv* and *wzz* genes. A duplex PCR method was developed for this purpose. The primer sequences (Table 1) for the searched genes were synthesized by Genomed (Warsaw, Poland). 

Multiplex PCR was conducted with the HotStart Taq *Plus* Master Mix Kit (Qiagen, Hilden, Germany). The reaction mixture contained 10 µL of HotStart Taq DNA Polymerase, 2 µL of CoralLoad Concentrate, 0.1 µL of each primer (with a final concentration of 0.5 µM), and approximately 100 ng of the isolated DNA (3 µL), supplemented with RNase-free water to a final volume of 20 µL. Three controls were established for each reaction, including two positive controls with DNA isolated from the reference strains (*Y. enterocolitica* 1B/O:8 [ACTT 23715], *Y. enterocolitica* 1A/O:8 [KU198401, NCBI]), and one negative control without DNA. Multiplex PCR was performed in a Mastercycler thermal cycler (Eppendorf) under conditions of initial denaturation at 95 °C for 5 min, followed by 30 cycles of denaturation at 94 °C for 30 s, primer annealing at 55 °C for 30 s, amplification at 72 °C for 60 s, and final chain synthesis at 72°C for 10 min. The products of triplex PCR were separated by electrophoresis on 2% agarose gel, and amplicon size was determined by comparison with the DNA mass standard (GeneRuler 100 bp DNA Ladder, Thermo Scientific, Waltham, MA, USA). The results were archived using the GelDoc system (Bio-Rad, Hercules, CA, USA). The composition and preparation of the reaction mix were not substantially modified for the duplex PCR. However, two different controls were established: a positive control (containing the DNA of a reference strain provided by Professor Michael Skurnik of the Department of Bacteriology and Immunology of the University of Helsinki) and a negative control without DNA. Duplex PCR was also performed in the Mastercycler thermal cycler (Eppendorf) under conditions of initial denaturation at 95 °C for 5 min, followed by 30 cycles of denaturation at 94 °C for 45 s, primer annealing at 60 °C for 60 s, amplification at 72 °C for 45 s, and final chain synthesis at 72 °C for 10 min. The products of triplex PCR were separated by electrophoresis on 2% agarose gel, and amplicon size was determined by comparison with the DNA mass standard (GeneRuler 100 bp DNA Ladder, Thermo Scientific). The results were archived using the GelDoc system (Bio-Rad).

### 2.3. High-Resolution Melting Analysis, Sequencing and Phylogenetic Analysis

Single nucleotide polymorphisms (SNPs) were identified in the analyzed amplicons with the use of a Type-it^®^ HRM^TM^ PCR Kit (Qiagen, Hilden, Germany) containing HRM PCR MasterMix and RNase-free water. The same primers were used for multiple PCR and high-resolution melting (HRM) analysis. The isolated DNA was diluted to a final concentration of approximately 50 ng/µL. The reaction mix was composed of 12.5 µL of the HRM PCR MasterMix, 1.75 µL of the primer mix, and 0.6 µL (50 ng) of the isolated DNA, supplemented with RNase-free water to a final volume of 25 µL. Four standards corresponding to regular genotypes [41] were used in each reaction. The reaction was carried out in the Rotor-Gene 6000 cycler (Corbett Life Science, Australia) with a dedicated HRM channel. The results were analyzed with the use of Rotor-Gene 6000 Series Software 1.7. A melting curve analysis was conducted within a temperature range of 65 °C to 90 °C, in steps of 0.1 °C, with a hold time of 2 s. 

The amplicons with sequences identical to the applied standards, as well as amplification products with melting curves that differed from the applied standards (variations), were directly sequenced to verify the results of the HRM analysis. All isolated strains of *Y. enterocolitica* contained only fragments of the y*stB* gene; therefore, the phylogenetic analysis was based on the nucleotide sequence of the y*stB* gene. A new primer pair with an amplification product of 263 bp was used for *ystB* (YSTB1—5′GGA CAC CGC ACA GCT TAT ATT TT3′, YSTB2—5′GCA CAG GCA CGA TTG CAA CA3′) to obtain a longer nucleotide sequence for the comparative analysis. 

The obtained product was cleaned using a CleanUp kit (A&A Biotechnology, Gdynia) according to the manufacturer’s instructions, and sequenced by Genomed (Warsaw, Poland). The nucleotide sequences of the *ystB* gene in the analyzed *Y. enterocolitica* strains were compared with previously described sequences in the BLAST v. 2.2.18 program [42]. Multiple sequence alignment was performed using the Clustal W algorithm [43] and Computational Evolutionary Biology Mega v. 10.0.4 software. Nucleotide sequences were visualized in BioEdit v. 7.2.0, and the phylogenetic tree was generated with the use of Mega v. 10.0.4.

### 2.4. Bioserotyping Analyses

The biotypes of the isolated *Y. enterocolitica* strains were determined with the use of the protocol described in Annex D of standard PN-EN ISO 10273 [44], which is based on the study by Wauters [45]. The analyzed *Y. enterocolitica* strains were biotyped based on their ability to ferment trehalose, xylose, and esculin, and to produce pyrazinamidase, Tween esterase, and indole. 

The examined strains were serotyped in the slide agglutination test, with live bacterial cells cultured on blood agar (Graso, Starogard Gdański) for 24 h as the antigen. The sera for somatic antigens O:3, O:5, O:8, O:9, and O:27 were supplied by Sifin Diagnostics (Berlin, Germany). Colonies cultured for 24 h on blood agar were suspended in a drop of 0.85% NaCl on a microscope slide. A drop of serum was applied to the slide and combined with the colony by use of an inoculation loop, and the mixture was shaken for 1 min. The result of the test was positive if agglutination occurred with one of the five tested sera. Strains that did not agglutinate with any of the sera were regarded as non-typable (NT).

### 2.5. Antimicrobial Sensitivity Analysis

A fresh bacterial culture was established on TSA medium, and incubated at a temperature of 28 °C for 24 h. Several emerged colonies were sampled and suspended in 0.85% saline solution, to obtain a suspension with a concentration equivalent to 0.5 McFarland standard units. Suspension density was determined nephelometrically with a colorimeter. A sterile swab was placed in the prepared suspension. Excess inoculum was removed by rotating and pressing the swab against the walls of the test tube, above the liquid. The suspension was streaked three times across the Mueller–Hinton medium, and the plate was rotated 60° each time. Antibiotic discs were placed on the medium within 15 min of establishing the bacterial culture. A stencil was employed to evenly distribute the discs. Each disc was gently pressed down using sterile tweezers, to ensure good contact with the medium. The plates were incubated at a temperature of 30 °C for 24 h, according to the guidelines of the Clinical and Laboratory Standards Institute (CLSI) [46]. After incubation, the zone of inhibition (including disc diameter) was measured in millimeters. A total of 13 chemotherapeutics were tested in the study: amoxicillin with clavulanic acid (30 µg), ampicillin (10 µg), cefotaxime (10 µg), ceftazidime (10 µg), cefalexin (30 µg), chloramphenicol (30 µg), ciprofloxacin (5 µg), gentamycin (10 µg), kanamycin (30 µg), nalidixic acid (30 µg), streptomycin (10 µg), trimethoprim-sulfamethoxazole (1.25/23.75 µg), and tetracycline (30 µg). The results were interpreted with the use of CLSI standards. 

## 3. Results 

### 3.1. Strain Isolation and Molecular Confirmation

A total of 372 rectal swabs were analyzed, including 262 swabs from free-living and 110 swabs from farmed fallow deer. Seven strains (2.7% of the analyzed samples) were isolated from free-living fallow deer. These strains were isolated from six (4.58%) out of the 131 examined animals. Strains from one animal were isolated from warm and cold cultures. The majority of strains (six strains, 85.7%) were isolated from warm culture. 

Two strains (1.8% of the analyzed samples) were isolated from farmed fallow deer. These strains were isolated from two (3.6%) out of the 55 examined animals, and both were isolated from warm culture. In multiplex PCR, products of size corresponding to *ystB* amplicons were observed in all isolated *Y. enterocolitica* strains. Amplification products characteristic of *ystA* or *ystC* were not observed in any of the examined strains. 

It should also be noted that five strains containing only amplicons of the *ail* gene with phenotypic traits similar to *Y. enterocolitica* were isolated from free-living fallow deer. However, the *ail* gene never occurs alone in *Y. enterocolitica* strains, and it is always accompanied by an *yst* gene. Strains with independently occurring *ail* genes were subjected to the API 20E biochemical test which revealed traits characteristic of *Y. kristensenii* and *Y. frederiksenii*/*Y. intermedia*. Amplicons of *inv* and *wzz* genes characteristic of *Y. pseudotuberculosis* strains were not identified in any of the analyzed animals. 

### 3.2. High-Resolution Melting, Sequencing and Phylogenetic Analysis

In the next stage of the study, *ystB*-positive *Y. enterocolitica* strains were analyzed using the HRM method. Melting curves were normalized in the 77.08–75.23 and 82.18–83.36 regions. The results were processed using the RotorGene-HRM software to classify the examined sequences based on sequence homology with reference genotypes. The SNP analysis of the *ystB* gene supported the classification of five strains into two different genotypes. Four nucleotide sequences of the *ystB* gene (strains 2D PSB, 30D ITC, 31D ITC, and 60D ITC) were characterized by 100% homology with *Y. enterocolitica* KJ592626 (NCBI) classified as genotype 3 (G3) based on the standard developed by Bancerz-Kisiel et al. [41]. One nucleotide sequence of the *ystB* gene (strain 57D ITC) was 100% identical to *Y. enterocolitica* KM253272 (NCBI) classified as genotype 1 (G1) by Bancerz-Kisiel et al. [41].

The SNP analysis of the *ystB* gene also revealed the presence of four strains (variations) with sequences that differed from the applied standards. Direct sequencing analysis of these four variations demonstrated that two strains (22 ITC and 39 ITC) were 100% identical to *Y. enterocolitica* KM253272 (NCBI), which were classified as genotype G1. The remaining two strains (2D ITC and 6D ITC) were characterized by 100% homology with *Y. enterocolitica* KJ592626 (NCBI) and were classified as genotype G3. All sequences from the strains isolated from fallow deer were deposited in the NCBI database.

**Figure 1:**
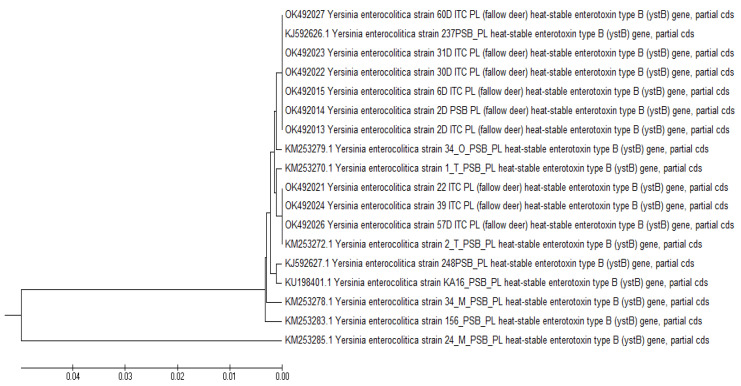
Phylogenetic analysis of *Y. enterocolitica* strains isolated from free-living and farmed fallow deer.

Detailed phylogenetic analysis of the strains isolated from free-living and farmed fallow dear based on the nucleotide sequences of the *ystB* gene revealed the presence of two phylogenetic groups (Figure 1). In the group of strains isolated from free-living fallow deer, genotype G3 was predominant (85.7%), and only one strain belonged to genotype G1 (14.3%). Both strains isolated from farmed deer also belonged to genotype G1. The phylogenetic analysis did not reveal nucleotide sequences characteristic of genotypes 2 or 4 (G2 or G4), or variation groups V1, V4, or V5, previously identified by Bancerz-Kisiel et al. in free-living animals [41]. 

### 3.3. Bioserotyping Analysis

The biotyping analysis revealed that all the isolated *Y. enterocolitica* strains belonged to biotype 1A. However, the serotyping analysis demonstrated that the majority of the isolated strains (seven strains, 77.8%) did not agglutinate with any diagnostic sera used in this study; these strains were classified as NT. One strain (11.1%) agglutinated with O:5 antigen serum, and one strain (11.1%) agglutinated with O:9 antigen serum. The agglutination slide test involving control strains with a known serotype produced correct results. Detailed results of the molecular assays are presented in Table 2, including the biotypes, serotypes, and genotypes of the isolated *Y. enterocolitica* strains. 

### 3.4. Antimicrobial Sensitivity Analysis

All molecularly confirmed strains of *Y. enterocolitica* were analyzed for sensitivity to chemotherapeutics. The highest number of strains (five strains, 55.55%) were sensitive to ciprofloxacin. Sensitivity to trimethoprim-sulfamethoxazole was noted in three (33.33%) out of the nine analyzed strains of *Y. enterocolitica*, and only one strain (11.11%) was sensitive to nalidixic acid. All strains were intermediately sensitive to tetracycline, and the great majority of strains (eight strains, 88.88%) also showed intermediate sensitivity to cefotaxime, ceftazidime, chloramphenicol, and nalidixic acid. Six strains (66.66%) were intermediately sensitive to trimethoprim-sulfamethoxazole, four strains (44.44%) to ciprofloxacin, three strains (33.33%) to kanamycin, and two strains (22.22%) to gentamycin. All analyzed *Y. enterocolitica* strains were resistant to amoxicillin with clavulanic acid, and to ampicillin, cefalexin, and streptomycin. Six strains (66.66%) were also resistant to gentamycin, and five strains (55.55%) to kanamycin. One strain was additionally resistant to cefotaxime, ceftazidime, and chloramphenicol. 

**Figure 2:**
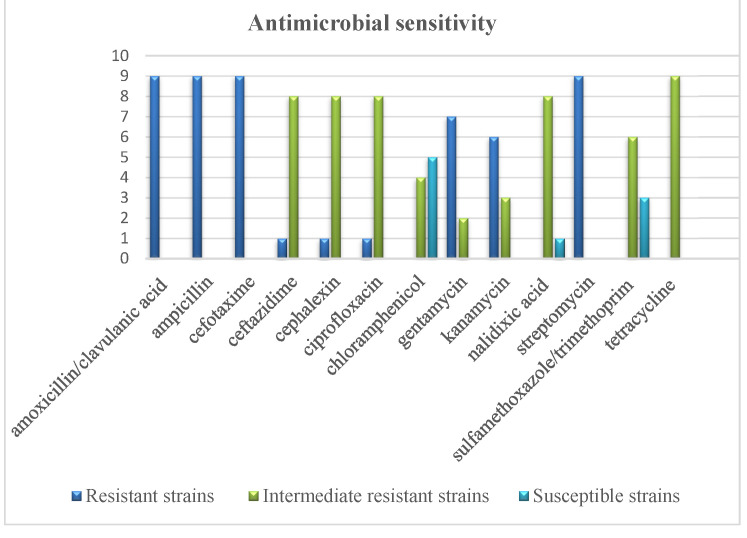
Antimicrobial sensitivity of *Y. enterocolitica* strains isolated from free-living and farmed fallow deer.

All *Y. enterocolitica* strains isolated from free-living and farmed fallow deer were multiple-drug-resistant and were insensitive to at least four of the tested chemotherapeutics. One *Y. enterocolitica* strain isolated from a free-living animal (2D ITC) was resistant to nine out of the 13 analyzed chemotherapeutics and was intermediately sensitive to the four remaining chemotherapeutics. Strain 30D ITC was resistant to six chemotherapeutics, intermediately sensitive to seven drugs, and was insensitive to any of the analyzed chemotherapeutics. Only one *Y. enterocolitica* strain isolated from a free-living animal (57D ITC) was sensitive to three out of the 13 tested chemotherapeutics (Figure 2). 

Differences were observed in the antibiotic-resistance profiles of two strains from the same animal isolated in two different cultures. The strain isolated in warm culture from a free-living animal (2D ITC) was resistant to cefotaxime, ceftazidime, and chloramphenicol, whereas the strain isolated in cold culture (2D PSB) was intermediately sensitive to these antibiotics. The strain isolated in warm culture was also intermediately sensitive to ciprofloxacin, whereas the strain isolated in cold culture was sensitive to this antibiotic. The chemotherapeutic-resistance profiles of *Y. enterocolitica* strains isolated from fallow deer are presented in Table 3.

## 4. Discussion

According to many studies, free-living animals are a reservoir of numerous pathogens, including *Y. enterocolitica* and *Y. pseudotuberculosis*, that are dangerous for humans and other animals [47]. Certain hunting techniques and the evisceration of game animals without adequate hygiene standards also promote the spread of pathogens in the environment, including carcass contamination with pathogenic strains of *Y. enterocolitica* and *Y. pseudotuberculosis* [48,49]. This observation gives serious cause for concern because the meat of fallow deer and other ruminants is often consumed raw. 

In the present study, multiplex PCR confirmed the identity of seven *Y. enterocolitica* strains (2.7%) isolated from free-living fallow deer, and two strains (1.8%) isolated from farmed deer. All isolated strains harbored fragments of the *ystB* gene that encodes the production of YstB enterotoxin. To date, the only study analyzing the prevalence of *Y. enterocolitica* strains in the Polish population of fallow deer was conducted by Syczyło et al. [24]. In that study, the identity of four *Y. enterocolitica* strains isolated from two out of 15 free-living fallow deer was confirmed with the use of molecular techniques. *Yersinia enterocolitica* strains were identified in 13.3% of the analyzed animals, which was a much higher percentage than that revealed in the current study. However, Syczyło et al. [24] studied a much smaller population of only 15 free-living fallow deer. All *Y. enterocolitica* strains isolated by Syczyło et al. [24] contained fragments of the *ystB* gene, consistent with the results of the current study. 

There have been no recent studies analyzing the prevalence of *Y. enterocolitica* and *Y. pseudotuberculosis* in fallow deer. The most recent studies in this field were conducted in the 1980s and 1990s. *Y. enterocolitica* and *Y. pseudotuberculosis* were not identified in any of the 42 samples acquired from farmed fallow deer in Australia [50]. Henderson [51] obtained 37 samples from farmed fallow deer in New Zealand and detected *Y. enterocolitica* in 24.3% of the animals. The isolated samples were not analyzed for the presence of virulence markers. *Yersinia pseudotuberculosis* has previously been isolated from free-living [52] and farmed fallow deer [53,54], but *Y. pseudotuberculosis* strains were not detected in the current study. *Yersinia enterocolitica* is generally very rarely found in Poland. This applies to human cases of infection with this bacterium reported to the Department of Epidemiology of Infectious Diseases and Surveillance in the National Institute of Public Health/National Institute of Hygiene in Poland, and also to animals. Although *Y. pseudotuberculosis* is most common in rodents, in the study by Platt-Samoraj et al. [55]_it was detected in only one out of 244 rodents tested. In comparison, *Y. enterocolitica* was isolated from 41 rodents.

It should be also mentioned that different species of wild animals inhabiting forests often rely on the same sources of drinking water, which is why the prevalence of *Y. enterocolitica* should be also examined in other free-living ruminants. Studies conducted in Poland by Bancerz-Kisiel et al. [56] and Syczyło et al. [24] revealed the presence of *Y. enterocolitica* strains in 12.5% and 21.6% of red deer and 4.16% and 9.4% of roe deer, respectively. In Switzerland, Joutsen et al. [20] isolated *Y. enterocolitica* from 10.0% of red deer, 13.0% of roe deer, 4.0% of alpine ibex, and 2.0% of chamois. In Italy, Avagnina et al. [57] isolated *Y. enterocolitica* from one out of 85 alpine ibex (1.2%), one out of 78 roe deer (1.3%), and one out of 56 red deer (1.8%). 

In the present study, analysis of nucleotide sequences in fragments of the *ystB* gene in nine *Y. enterocolitica* strains isolated from free-living and farmed fallow deer revealed the presence of phylogenetic groups G1 and G3. Most strains isolated from free-living animals belonged to genotype G3, whereas both strains isolated from farmed fallow deer belonged to genotype G1. Strains belonging to genotype G3 isolated from beaver and wild boars were reported by Platt-Samoraj et al. [58] and Bancerz-Kisiel et al. [41]. Strains belonging to genotype G1 isolated from red deer were described by Bancerz-Kisiel et al. [41]. Sequences of the *ystB* gene identical to genotype G1 were also reported in *Y. enterocolitica* strains isolated from food in Korea (GenBank CP009456.1), and from humans in Great Britain (GenBank HF571988.1) and Japan (GenBank D88145.1). 

In the present study, all isolated strains of *Y. enterocolitica* belonged to biotype 1A. Similar results were reported by Syczyło et al. [24], who analyzed four *Y. enterocolitica* strains isolated from 15 free-living fallow deer. Henderson [51] analyzed nine *Y. enterocolitica* strains isolated from fallow deer farmed in New Zealand, and only three of these strains belonged to biotype 1A. Biotype 1A strains of *Y. enterocolitica* have also been isolated from other species of free-living ruminants, including red deer, roe deer, and chamois [20,24,56]. Biotype 1A strains are generally regarded as non-pathogenic. However, they have increasingly been isolated from clinical cases involving humans [59,60], which could suggest that the pathogenic potential of *Y. enterocolitica* strains cannot be reliably determined based on biotype alone [61].

In the current study, the vast majority of *Y. enterocolitica* strains were classified as NT. Only two strains isolated from free-living fallow deer were serologically typed; one strain was classified as serotype O:5 and another as serotype O:9. Serotyping differences were also reported by Syczyło et al. [24], who found that two out of four *Y. enterocolitica* strains isolated from free-living fallow deer were classified as NT, one strain was classified as serotype O:5, and one strain as serotype O:9. *Yersinia enterocolitica* strains were not serotyped by other authors who characterized strains isolated from free-living and farmed fallow deer [50,51]. *Y. enterocolitica* strains isolated from other species of free-living ruminants in Poland were characterized by similar variations in serotype profiles to those described in this study [24,56]. 

In the present study, all strains were resistant to amoxicillin with clavulanic acid, and to ampicillin, cefalexin, and streptomycin. In the study of Joutsen et al. [20], all *Y. enterocolitica* strains isolated from various species of free-living ruminants were also resistant to ampicillin, amoxicillin with clavulanic acid, and cefalotin (a first-generation cephalosporin antibiotic, analogous to cefalexin). In the current study, 66.66% of the examined strains were also resistant to gentamycin, and 55.55% of strains were resistant to kanamycin. In contrast, none of the *Y. enterocolitica* strains isolated by Joutsen et al. [20] from free-living ruminants were resistant to streptomycin or kanamycin. All strains isolated in the present study were intermediately sensitive to tetracycline. In the study by Bonke et al. [62], all *Y. enterocolitica* strains isolated from wild boars in Germany were sensitive to tetracycline. 

A high percentage (55.55%) of the strains isolated in this study were sensitive to ciprofloxacin, which is a second-generation quinolone antibiotic. Sensitivity to ciprofloxacin was reported in 100% of the strains examined by Bonke et al. [62] and Szych et al. [63], and in 99.0% of the strains analyzed by Perkowska et al. [64]. These observations suggest that *Y. enterocolitica* strains are highly sensitive to ciprofloxacin, an antibiotic that is widely used in the treatment of yersiniosis. In the present study, only three out of the nine isolated *Y. enterocolitica* strains were also sensitive to trimethoprim-sulfamethoxazole, whereas 100% of the strains analyzed by Bonke et al. [62] were sensitive to this antibiotic. Sensitivity to trimethoprim-sulfamethoxazole was also reported in 97.4% of *Y. enterocolitica* strains studied by Szych et al. [63] and in 71.8% of *Y. enterocolitica* strains isolated by Perkowska et al. [64].

The results of the present study and the findings of other authors indicate that *Y. enterocolitica* strains have varied sensitivity to selected chemotherapeutics. It should also be noted that all the analyzed strains were multiple-drug-resistant. *Yersinia enterocolitica* strains isolated from free-living and farmed fallow deer were resistant to at least four of the tested chemotherapeutics. One strain was resistant to nine out of the 13 analyzed chemotherapeutics, and intermediately sensitive to the four remaining drugs. Only one *Y. enterocolitica* strain isolated from a free-living animal was sensitive to three out of the 13 examined antibiotics, whereas the remaining strains were sensitive to only one drug or were not sensitive to any of the chemotherapeutics used. It should also be stated that the chemotherapeutic-resistance profiles of *Y. enterocolitica* strains isolated from farmed fallow deer did not differ from the profiles found in free-living fallow deer. They were resistant to ampicillin, cefalexin, gentamycin, kanamycin, streptomycin, and amoxicillin with clavulanic acid, they showed intermediate resistance to cefotaxime, ceftazidime, chloramphenicol, nalidixic acid, trimethoprim/sulfamethoxazole, tetracycline, and they were susceptible to ciprofloxacin. This was the most common profile, demonstrated in four strains, while the remaining chemotherapeutic-resistance profiles were found in single strains.

## 5. Conclusions

The results of this study indicate that *Y. enterocolitica* can be carried by free-living and farmed fallow deer, making them potential sources of infection for consumers of game meat. This observation gives some cause for concern because the meat of fallow deer and other ruminants is often consumed semi-raw (steak) or raw (tartar steak). All *Y. enterocolitica* strains isolated from fallow deer were multiple-drug-resistant, which could pose a public health threat. 

## Figures and Tables

**Table 1:** Primer sequences for identifying fragments of *ail, ystA*, *ystB*, and *ystC* genes in *Y. enterocolitica*, and fragments of *inv* and *wzz* genes in *Y. pseudotuberculosis*.

Gene	Primer Sequence	Product Size	Reference
*ail*	5’TGGTTATGCGCAAAGCCATGT3’ 5’TGGAAGTGGGTTGAATTGCA3’	356 bp	[39]
*ystA*	5’GTCTTCATTTGGAGGATTCGGC3’ 5’AATCACTACTGACTTCGGCTGG3’	134 bp	[40]
*ystB*	5’TGTCAGCATTTATTCTCAACT3’ 5’GCCGATAATGTATCATCAAG3’	180 bp	[40]
*ystC*	5’TCGACAAGTGAGTGACGGAG3’ 5’CCCTTACTCGCGACGAAATA3’	284 bp	[40]
*inv*	5’GCAGAATTCGGATACCCAGCACCATGACT3’ 5’GCAGGATCCAGCCATGAACATTCCACA3’	689 bp	Skurnik *
*wzz*	5’GGTGATGAGCAAGTTCAAG3’ 5’GCTAAATCCACTGCTCGCTG3’	418 bp	[35]

* Sequences provided by Professor Michael Skurnik of the Department of Bacteriology and Immunology of the University of Helsinki.

**Table 2:** Characteristics of *Y. enterocolitica* strains isolated from free-living and farmed fallow deer.

Source	Strain Number	Virulence Markers				Genotype	Biotype	Serotype
		*ail*	*ystA*	*ystB*	*ystC*			
Free-living fallow deer	2D ITC	−	−	+	−	G3	1A	NT
	2D PSB	−	−	+	−	G3	1A	NT
	6D ITC	−	−	+	−	G3	1A	NT
	30D ITC	−	−	+	−	G3	1A	NT
	31D ITC	−	−	+	−	G3	1A	O:9
	57D ITC	−	−	+	−	G1	1A	O:5
	60D ITC	−	−	+	−	G3	1A	NT
Farmed fallow deer	22 ITC	−	−	+	−	G1	1A	NT
	39 ITC	−	−	+	−	G1	1A	NT

**Table 3:** Antibiotic resistance profiles of *Y. enterocolitica* strains isolated from free-living and farmed fallow deer.

Source	Sample Number	Amoxicillin/Clavulanic Acid	Ampicillin	Cefalexin	Cefotaxime	Ceftazidime	Chloramphenicol	Ciprofloxacin	Gentamycin	Kanamycin	Nalidixic Acid	Streptomycin	Trimethoprim/Sulfamethoxazole	Tetracycline
Free-living fallow deer	2D ITC	R	R	R	R	R	R	I	R	R	I	R	I	I
	2D PSB	R	R	R	I	I	I	S	R	R	I	R	I	I
	6D ITC	R	R	R	I	I	I	S	R	R	I	R	I	I
	30D ITC	R	R	R	I	I	I	I	R	R	I	R	I	I
	31D ITC	R	R	R	I	I	I	I	R	I	I	R	S	I
	57D ITC	R	R	R	I	I	I	S	I	I	S	R	S	I
	60D ITC	R	R	R	I	I	I	I	I	I	I	R	S	I
Farmed fallow deer	22 ITC	R	R	R	I	I	I	S	R	R	I	R	I	I
	39 ITC	R	R	R	I	I	I	S	R	R	I	R	I	I

R—resistant; I—intermediate resistant; S—sensitive.

## Data Availability

Not applicable.
